# JAK2 tyrosine kinase mediates integrin activation induced by CXCL12 in B-cell chronic lymphocytic leukemia

**DOI:** 10.18632/oncotarget.5196

**Published:** 2015-09-10

**Authors:** Alessio Montresor, Lara Toffali, Michela Mirenda, Antonella Rigo, Fabrizio Vinante, Carlo Laudanna

**Affiliations:** ^1^ Department of Pathology and Diagnostics, Division of General Pathology, Laboratory of Cell Trafficking and Signal Transduction, University of Verona, Verona 37134, Italy, EU; ^2^ The Center for Biomedical Computing (CBMC), University of Verona, Verona 37134, Italy, EU; ^3^ Department of Medicine, Section of Hematology, Cancer Research & Cell Biology Laboratory, University of Verona, Verona 37134, Italy, EU

**Keywords:** chronic lymphocytic leukemia, adhesion, integrin activation, chemokines, JAKs

## Abstract

Chemokines participate to B-cell chronic lymphocytic leukemia (B-CLL) pathogenesis by promoting cell adhesion and survival in bone marrow stromal niches and mediating cell dissemination to secondary lymphoid organs. In this study we investigated the role of JAK protein tyrosine kinases (PTK) in adhesion triggering by the CXC chemokine CXCL12 in normal versus CLL B-lymphocytes. We demonstrate that CXCL12 activates JAK2 in normal as well as CLL B-lymphocytes, with kinetics consistent with rapid adhesion triggering. By using complementary methodologies of signal transduction interference, we found that JAK2 mediates CXCL12-triggered activation of lymphocyte function-associated antigen-1 (LFA-1) and very late antigen-4 (VLA-4) integrins. We also show that JAK2 mediates the activation of the small GTP-binding protein RhoA, in turn controlling LFA-1 affinity triggering by CXCL12. Importantly, comparative analysis of 41 B-CLL patients did not evidence JAK2 functional variability between subjects, thus suggesting that JAK2, differently from other signaling events involved in adhesion regulation in B-CLL, is a signaling molecule downstream to CXCR4 characterized by a conserved regulatory role. Our results reveal JAK2 as critical component of chemokine signaling in CLL B-lymphocytes and indicate JAK inhibition as a potentially useful new pharmacological approach to B-CLL treatment.

## INTRODUCTION

B-cell chronic lymphocytic leukemia (B-CLL) is a common, rather heterogeneous, leukemia, characterized by progressive accumulation of functionally incompetent B-lymphocytes in the bone marrow, blood and lymphoid organs [1]. B-CLL cells are primarily characterized by loss of appropriate apoptosis, although this characteristic is lost when B-CLL cells are removed from the host, clearly suggesting a critical role for micro environmental factors and/or adhesive stromal interactions [2–4]. Several chromosome, gene and signaling abnormalities have been described and differently correlated to disease progression [5]. Decrease of activity of the tumor suppressor gene p53 and other pro-apoptotic proteins, such as BAD and PKR, has been variably reported. On the other side, increase in pro-survival pathways regulated by BCR, TCL1, NF-*k*B and PIP3K has been shown. Furthermore, protein tyrosine kinases (PTKs), such as ZAP-70 and Syk, have a variable role in driving survival pathways and have been considered to be B-CLL prognostic markers [6–8]. Importantly, Bruton's tyrosine kinase (BTK) has been shown to play a role in survival and is now a very promising pharmacological target for B-CLL treatment [9].

B-CLL cells also display altered mechanisms of integrin activation and lymphoid tissue dissemination in response to homeostatic chemokines [10, 11]. We have previously reported that, in CLL B-lymphocytes, the rho-signaling module of LFA-1 affinity triggering we have described [12] is abnormal. Indeed, by analyzing 31 B-CLL patients, we observed a consistent variability in the regulatory role of molecules belonging to the rho-module of integrin activation, and this allowed grouping the patients in two categories, characterized by not conserved roles of signaling molecules regulating LFA-1 activation by the CXC chemokine CXCL12 [13]. Overall, CLL B-lymphocytes experience an environment-dependent unbalance between pro- and anti-apoptotic signaling mechanisms, along with anomalies in inside-out signaling events controlling integrin activation and adhesion. The exact means by which B-CLL genetic alterations lead to leukemogenesis is still unclear and this hampers the possibility of reliable prognosis and affects the outcome of current treatments, which fail to eradicate the disease and remain unsatisfactory.

Chemokines likely play a relevant role in B-CLL biology, due to their anti-apoptotic effects and their ability to direct cell migration and tissue homing [10, 14]. Thus, defining chemokine-triggered signaling mechanisms can be of help in B-CLL therapy. At least 67 intracellular signaling proteins have been reported to control integrin activation by chemokines [15], although only few have been studied according to previously formalized *four criteria* [15]. Very recently, we have demonstrated the central role of Janus tyrosine kinases (JAKs) in CXCL12-activated inside-out signaling controlling integrin affinity modulation and *in vivo* homing of human primary T-lymphocytes [16]. However, the role of JAK PTKs in integrin activation by chemokines in CLL B-lymphocytes is unknown. To address the role of JAK PTKs in B-CLL, we performed a comparative analysis by applying a previously developed experimental approach [16]. We show that CXCL12 activates JAK2 in CLL as well as in normal B-lymphocytes with corresponding mechanisms and with kinetics consistent with rapid integrin activation. JAK2 inhibition prevents LFA-1 and VLA-4-mediated rapid adhesion. Moreover, we found that JAK2 controls chemokine-driven LFA-1 conformational changes leading to affinity increase. Finally, signal transduction analysis showed that JAK2 is the upstream activator of the small GTPase RhoA also in neoplastic CLL B-lymphocytes. Importantly, no JAK-independent adhesion phenotype was found among 41 studied B-CLL patients. Taken together, the data identify JAK2 as a conserved major signal transduction mechanism involved in CXCL12 signaling in B-CLL and suggest that inhibition of JAK PTKs could be a new valuable pharmacological approach to B-CLL therapy.

## RESULTS

### JAK2 is activated by CXCL12 and mediates integrin affinity triggering and dependent adhesion in normal B-lymphocytes

**Figure 1:**
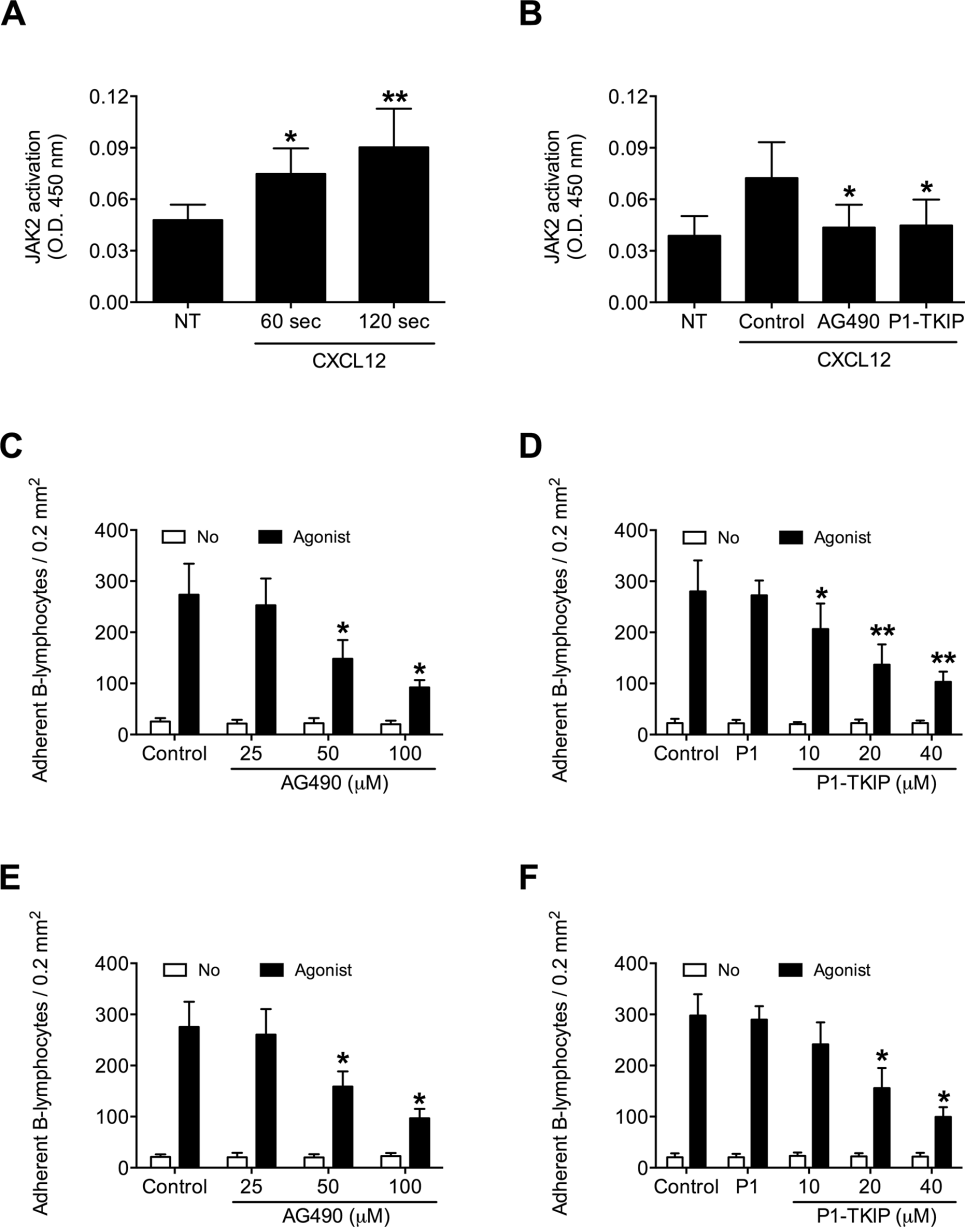
JAK2 is activated by CXCL12 and mediates B-lymphocyte static adhesion to ICAM-1 and VCAM-1 **A.** Cells were treated with buffer (NT) or CXCL12 0.5 μM for indicated times. Mean ± SD. *P < 0.01; **P < 0.001, versus NT. **B.** Cells were treated for 1 h with vehicle (NT and Control), AG490 100 μM, or P1-TKIP 40 μM, and stimulated with CXCL12 0.5 μM (Agonist) for 120 sec. Mean ± SD. *P < 0.01, versus Control. Static adhesion to ICAM-1 **C–D** or VCAM-1 **E–F.** Cells were treated for 1 h with vehicle (Control) or the indicated doses of AG490 (C-E), or with vehicle (Control), P1 40 μM or the indicated doses of P1-TKIP (D–F), and stimulated with buffer (No) or CXCL12 0.5 μM (Agonist). Mean ± SD. *P < 0.001, versus Control (C-E). Mean ± SD. *P < 0.001, versus P1 (D–F). Data are average of *n* = 10 independent experiments in duplicate using B-cells isolated from 10 different healthy donors.

**Figure 2:**
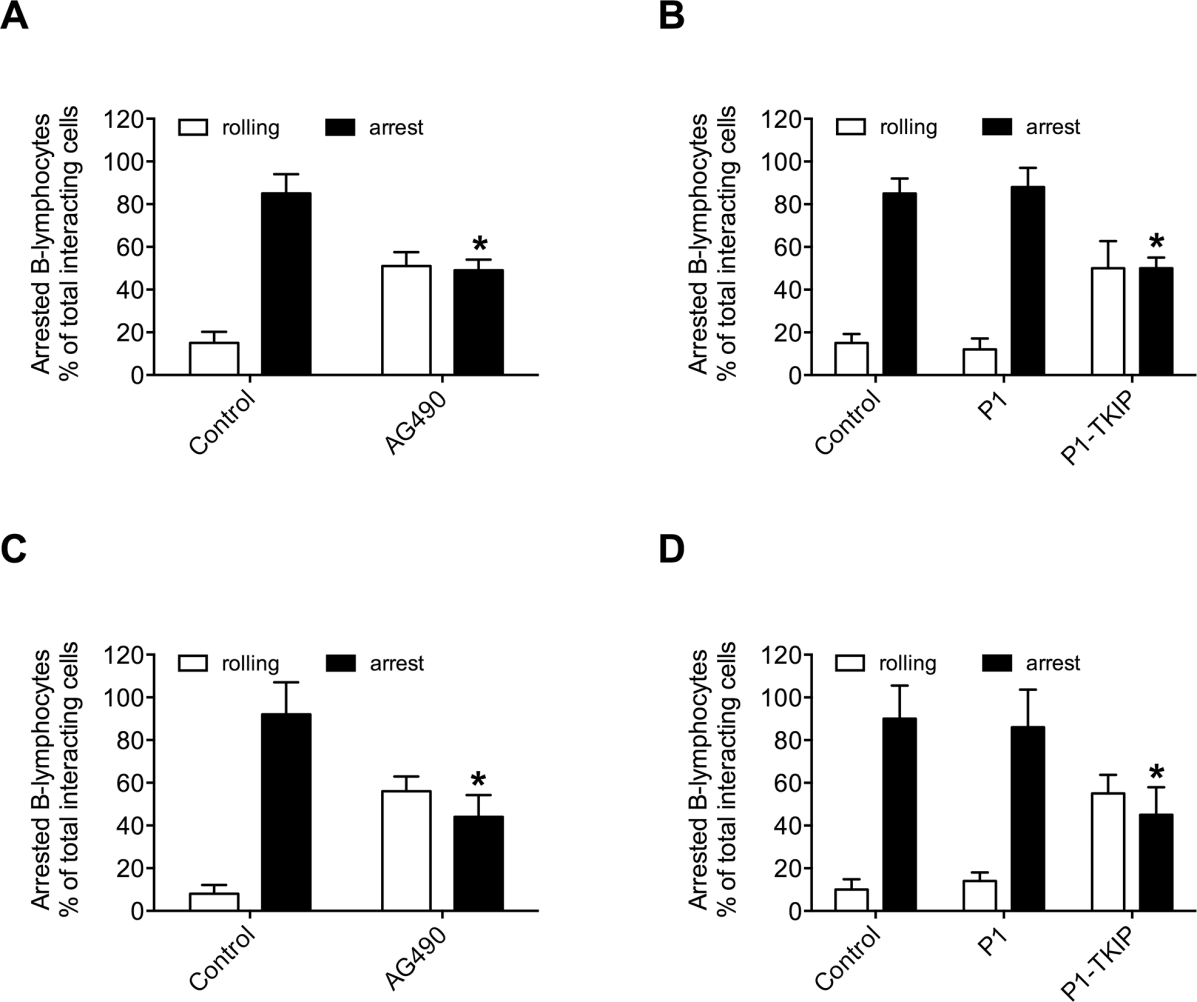
JAK2 mediates CXCL12-mediated B-lymphocyte underflow adhesion to ICAM-1 and VCAM-1 Underflow adhesion to ICAM-1 **A–B** or VCAM-1 **C–D.** Cells were treated for 1 h with vehicle (Control) or with AG490 100 μM (A–C), or P1 or P1-TKIP 40 μM (B–D). Mean ± SD. *P < 0.001, versus Control (A–C). Mean ± SD. *P < 0.001, versus P1 (B–D). Data are average of *n* = 10 independent experiments using B-cells isolated from 10 different healthy donors.

**Figure 3:**
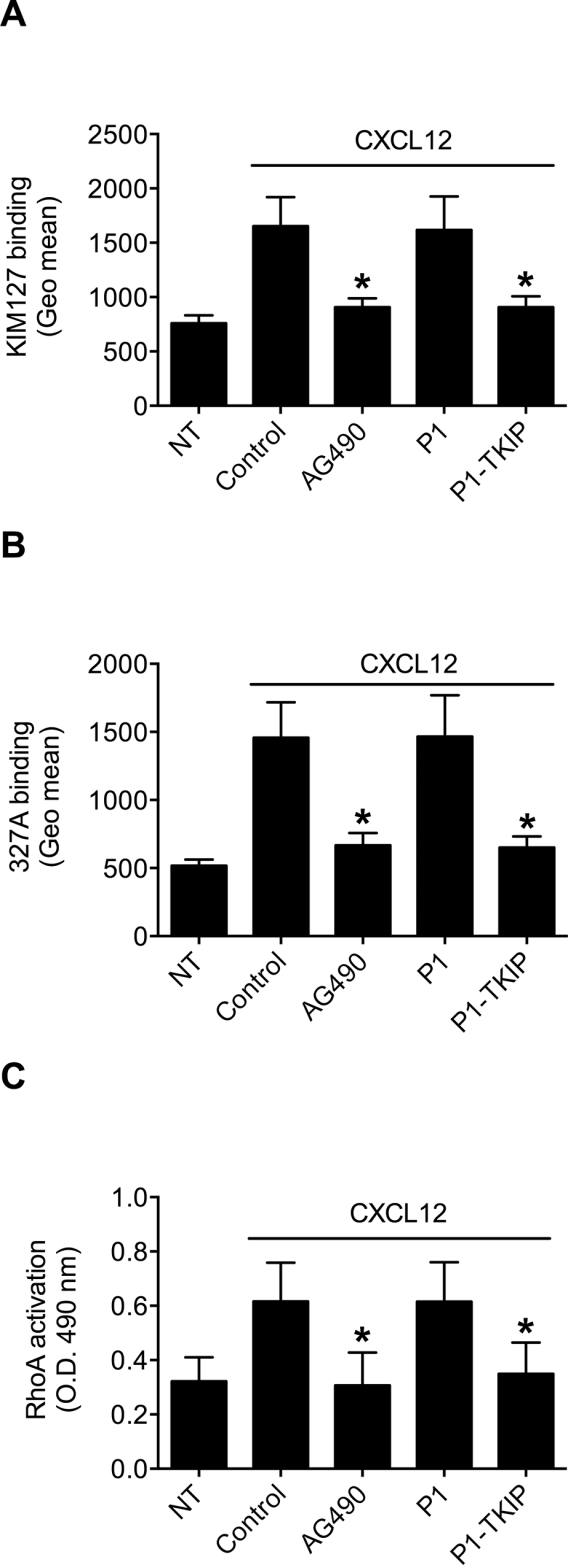
JAK2 mediates LFA-1 affinity triggering and RhoA activation by CXCL12 in B-lymphocytes **A.** KIM127 or **B** 327A staining; cells were treated for 1 h with vehicle (NT and Control), AG490 100 μM, P1 or P1-TKIP 40 μM, and stimulated with CXCL12 0.5 μM for 120 sec. Mean ± SD. *P < 0.001, versus Control or P1. **C.** RhoA activation; cells were treated and stimulated as in (A–B). Mean ± SD. *P < 0.001, versus Control or P1. Data are average of *n* = 10 independent experiments (in duplicate for RhoA activation) using B-cells isolated from 10 different healthy donors.

To investigate JAK2 involvement in chemokine-induced integrin triggering in B-lymphocytes we first performed a biochemical analysis and found that CXCL12 triggers in normal B-lymphocytes JAK2 tyrosine auto-phosphorylation on Tyr 1007 and 1008, with kinetics consistent with rapid adhesion triggering (Figure 1A). We then evaluated JAK2 involvement in integrin activation by taking advantage of two different approaches, including the known JAKs inhibitor Tyrphostin AG490 and the more selective Penetratin-1 (P1)-fusion peptide P1-TKIP we recently characterized [16]. Biochemical analysis showed that JAK2 auto-phosphorylation was strongly prevented by both AG490 and P1-TKIP peptide (Figure 1B), as expected. The P1 control peptide was totally ineffective (data not shown). Importantly, pretreatment with AG490 or with P1-TKIP peptide inhibited, in a dose-dependent manner, CXCL12-triggered B-lymphocyte adhesion to ICAM-1 (Figures 1C and 1D) and to VCAM-1 (Figures 1E and 1F). To further confirm the previous results under more physiological conditions, we evaluated the effect of JAK2 inhibition on CXCL12-triggered arrest in underflow adhesion assays under physiological shear stress conditions. Pretreatment of B-lymphocytes with AG490 or P1-TKIP peptide significantly reduced CXCL12-triggered rapid arrest on ICAM-1. Moreover, inhibition of arrest was accompanied by a concomitant increase of rolling cells, as expected (Figures 2A and 2B). A similar cell behavior was observed in underflow adhesion to VCAM-1 (Figures 2C and 2D). The P1 control peptide was ineffective. To conclusively characterize the regulatory role of JAK2 in integrin activation by chemokines in normal B-lymphocytes, we assessed the effect of JAK2 blockade on CXCL12-induced LFA-1 conformational changes leading to progressive affinity increase. JAK2 inhibition almost completely prevented CXCL12-induced transition of LFA-1 to extended conformations recognized by the reporter monoclonal antibodies KIM127 (Figure 3A and Supplementary Figure S1A) and 327A (Figure 3B and Supplementary Figure S1B), detecting low-intermediate and high affinity states, respectively. Taken together, these findings, obtained under physiological conditions, show that JAK2 mediates CXCL12-induced intracellular signaling leading to LFA-1 and VLA-4 affinity activation and dependent arrest of normal B-lymphocytes, thus showing that JAK2 is likely an important regulator of B-lymphocyte trafficking.

### JAK2 down-modulation prevents B-lymphocyte adhesion and LFA-1 affinity triggering by CXCL12

**Figure 4:**
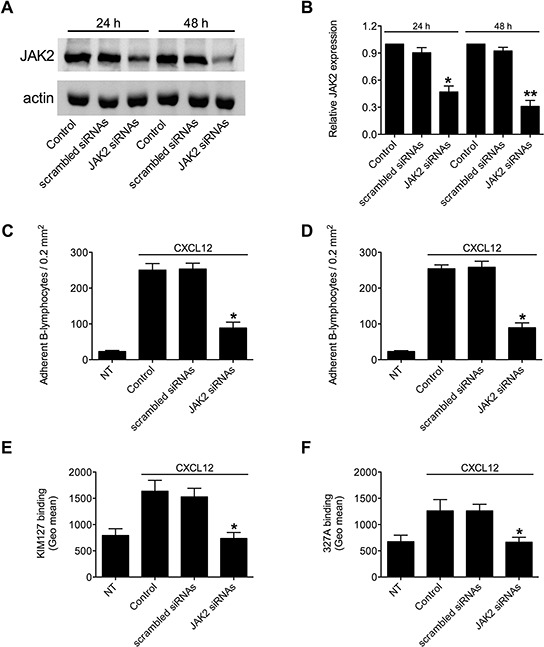
JAK2 down-modulation by siRNAs prevents chemokine-induced adhesion and LFA-1 affinity triggering **A.** Western blot of total cell lysates of B-lymphocytes not treated (Control), nucleoporated with a pool of 4 scrambled or JAK2-specific siRNAS and kept in culture for 24 h or 48 h. Shown is protein content compared to actin; one representative experiment of three. **B.** Quantification of immunoreactive bands of *n* = 3 independent experiments. The y-axis represents the relative JAK2/actin protein ratio normalized to the Control value. Mean ± SD. *P < 0.05; **P < 0.01, versus scrambled siRNAs. Static adhesion to ICAM-1 **C** or VCAM-1 **D.** Cells were treated as in (A) and after 48 h were collected and stimulated with CXCL12 0.5 μM for 120 sec. Mean ± SD. *P < 0.001, versus scrambled siRNAs; data are average of *n* = 3 independent experiments in triplicate. **E.** KIM127 or **F** 327A staining: cells were treated as in (A) and after 48 h were collected and stimulated with CXCL12 0.5 μM for 120 sec. Mean ± SD. *P < 0.01, versus scrambled siRNAs; data are average of *n* = 3 independent experiments.

To corroborate the above results, we applied a siRNA-based approach, we previously validated in T-lymphocytes [16]. A pool of four different siRNAs specific for JAK2 effectively reduced the expression level of JAK2 (Figure 4A); 48 h treatment was maximally effective, with mean decrease of 65%, compared to scrambled siRNAs (Figure 4B), and was, then, chosen as standard time of treatment. B-lymphocytes showing reduced expression of JAK2 displayed an impaired CXCL12-triggered adhesion to both ICAM-1 and VCAM-1 (Figures 4C and 4D, respectively). Moreover, we observed an almost complete blockade of CXCL12-triggered LFA-1 transition to low-intermediate (Figure 4E and Supplementary Figure S1C) and to high (Figure 4F and Supplementary Figure S1D) affinity states. Overall, the siRNAs data are in keeping with the inhibitors data and fully confirmed the regulatory role of JAK2 in integrin affinity triggering and dependent adhesion by CXCL12 in B-lymphocytes.

### JAK2 controls CXCL12-triggered RhoA activation in normal B-lymphocytes

The involvement of JAK2 in integrin affinity triggering and dependent adhesion by CXCL12 prompted us to investigate whether the activation of the rho-module of integrin affinity modulation, we formerly characterized in T-lymphocytes [12], was controlled by JAK PTKs also in B-lymphocytes. Notably, in the context of the rho-module of integrin affinity triggering by chemokines, we have previously reported that the small GTPase RhoA, along with its effector PLD1, is the most conserved signaling event in CLL B-lymphocytes [13]. Thus, to be compliant with the comparative investigation, we focused the analysis on RhoA activation. JAK2 inhibition by AG490 and P1-TKIP peptide completely prevented CXCL12-triggered GTP loading of RhoA thus blocking its activation (Figure 3C). The P1 control peptide was completely ineffective. Altogether, these data establish JAK2 as critical mediator of RhoA activation by CXCL12 in primary normal B-lymphocytes.

### JAK2 is a conserved CXCL12-triggered signaling event in CLL B-lymphocytes

**Figure 5:**
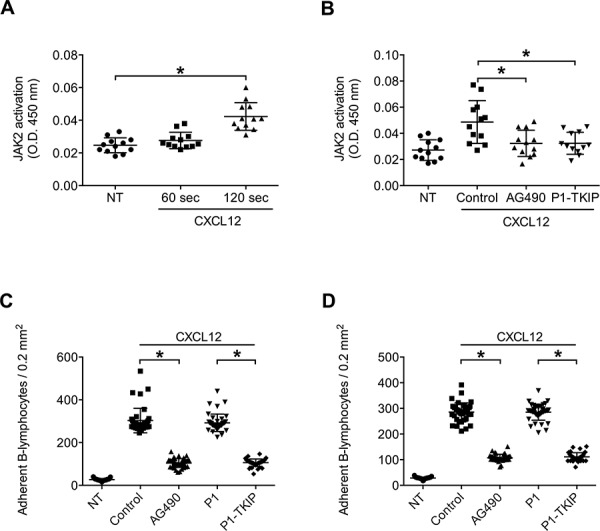
JAK2 is activated by CXCL12 and mediates CLL B-lymphocytes static adhesion to ICAM-1 and VCAM-1 **A.** Cells were treated with buffer (NT) or CXCL12 0.5 μM for indicated times. Mean ± SD. *P < 0.01; **P < 0.001, versus NT; *n* = 12 (patients samples) independent experiments in duplicate. **B.** Cells were treated for 1 h with vehicle (NT and Control), AG490 100 μM, or P1-TKIP 40 μM, and stimulated with CXCL12 0.5 μM for 120 sec. Mean ± SD. *P < 0.01, versus Control; *n* = 12 (patients samples) independent experiments in duplicate. Static adhesion to ICAM-1 **C** or VCAM-1 **D.** Cells were treated for 1 h with vehicle (NT and Control) or with AG490 100 μM, P1 or P1-TKIP 40 μM and stimulated as in (B). Mean ± SD. *P < 0.001, versus Control or P1; data are average of *n* = 41 independent experiments in triplicate using B-CLL cells isolated from 41 patients.

We, then, proceeded to investigate whether JAK2 was functional in neoplastic B-lymphocytes isolated from patients with a diagnosis of B-CLL. To study JAK2 role in chemokine signaling in B-CLL, and to evidence possible individual variability, we performed a comparative analysis of neoplastic B-lymphocytes isolated from a total of 41 B-CLL patients, by evaluating the activation state of JAK2 and its regulatory role in chemokine-induced LFA-1- and VLA-4-dependent adhesion. As shown in Figure 5A, JAK2 was rapidly phosphorylated on Tyr 1007 and 1008 upon CXCL12 stimulation, with kinetics consistent with rapid integrin triggering. The data from the analyzed patients were averaged and showed an apparent slower kinetics of JAK2 activation with respect to normal B-lymphocytes. However, no patient with defective JAK2 activation by CXCL12 was found. Notably, as shown in Figure 5B, cell treatment with either AG490 or P1-TKIP blocking peptide almost completely inhibited JAK2 phosphorylation of regulatory tyrosine residues by CXCL12. Thus, at biochemical level, JAK2 appears undergoing in CLL B-lymphocytes the same regulation by CXCL12 observed in normal cells with no variability between the studied patients.

### JAK2 mediates CXCL12-triggered integrin activation and dependent adhesion in CLL B-lymphocytes

**Figure 6:**
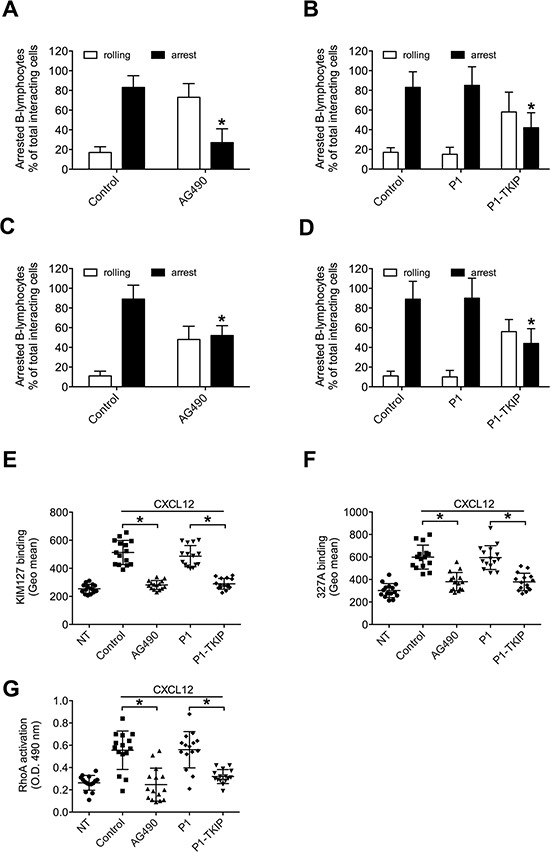
JAK2 mediates underflow adhesion, LFA-1 affinity triggering and RhoA activation by CXCL12 in CLL B-lymphocytes Underflow adhesion to ICAM-1 **A–B** or VCAM-1 **C–D.** Cells were treated for 1 h with vehicle (Control), AG490 100 μM, P1 or P1-TKIP 40 μM. Mean ± SD. *P < 0.01, versus Control; *n* = 5 (patients samples) independent experiments. **E.** KIM127 or **F** 327A staining: cells were treated for 1 h with vehicle (NT and Control), AG490 100 μM, P1 or P1-TKIP 40 μM, and stimulated with CXCL12 0.5 μM for 120 sec. Mean ± SD. *P < 0.001, versus Control or P1; *n* = 15 (patients samples) independent experiments. **G.** RhoA activation: cells were treated and stimulated as in (E–F). Mean ± SD. *P < 0.001, versus Control or P1; *n* = 15 (patients samples) independent experiments in duplicate.

We then investigated JAK2 role in CXCL12-induced integrin activation in CLL B-lymphocytes. Pretreatment with AG490 and P1-TKIP JAK2 inhibitory peptide blocked CXCL12-triggered static adhesion of CLL B-lymphocytes to both ICAM-1 and VCAM-1 (Figures 5C and 5D). The P1 control peptide was ineffective. Furthermore, in underflow adhesion assays, pretreatment with AG490 inhibited CXCL12-mediated rapid arrest on both ICAM-1 (Figure 6A) and VCAM-1 (Figure 6C), with a corresponding increase of rolling, as expected. Similar results were obtained by using the P1-TKIP JAK2 blocking peptide whereas the P1 control peptide was ineffective (Figures 6B and 6D). To further confirm JAK2 involvement in integrin activation in CLL B-lymphocytes we evaluated LFA-1 affinity triggering. Pretreatment of CLL B-lymphocytes with AG490 or P1-TKIP peptide strongly prevented CXCL12-triggered LFA-1 transition to low-intermediate (Figure 6E and Supplementary Figure S1E) and to high affinity states induced by CXCL12 (Figure 6F and Supplementary Figure S1F). These data are in keeping with data showing blockade of arrest underflow and show the functional role of JAK2 in chemokine-induced integrin-dependent adhesion in B-CLL lymphocytes.

### JAK2 controls CXCL12-triggered RhoA activation in CLL B-lymphocytes

As previously demonstrated [13], the small GTPase RhoA plays a critical and conserved role in the CXCL12-induced inside-out signaling leading to LFA-1 affinity up regulation in CLL B-lymphocytes, but the mechanism of RhoA activation by CXCL12 in B-CLL is unknown. Moreover, considering the individual signaling variability previously evidenced in CLL B-lymphocytes isolated from different patients [13], it was important to verify whether the regulatory relationship between JAK2 and RhoA observed in normal B-lymphocytes was also conserved in CLL B-lymphocytes. As shown in Figure 6G, in the group of analyzed patients JAK2 inhibition by AG490 and P1-TKIP peptide almost completely prevented CXCL12-triggered RhoA activation, whereas the P1 control peptide was ineffective. Collectively, our data demonstrate that JAK2 plays a central function in chemokine-mediated CLL B-lymphocyte adhesion, by regulating RhoA-mediated inside-out signal transduction controlling integrin affinity increase and dependent adhesion.

## DISCUSSION

Anomalous dissemination and survival of CLL B-lymphocytes is related to the activity of homeostatic chemokines, such as CXCL12, produced by stromal cells. CXCR4-CXCL12 axis is a major signal transduction mechanism controlling integrin activation, adhesion and migration, key events regulating accumulation in stromal niches and survival of CLL B-lymphocytes. In keeping with this observation, the expression of the CXCL12 receptor CXCR4 is higher in CLL B-cells than in normal B-lymphocytes [17], indicating the relevant regulatory role of CXCR4-mediated signaling in B-CLL progression and suggesting that the pharmacological targeting of CXCR4-triggered signal transduction can be a valuable approach to B-CLL therapy. In this study we analyzed the pro-adhesive signaling mechanisms triggered by CXCL12 in neoplastic CLL B-lymphocytes with the purpose of identifying novel targets of pharmacological treatment in B-CLL. Our findings can be summarized as follows: a- JAK2 is activated by CXCL12 and mediates integrin activation in primary normal as well as in neoplastic CLL B-lymphocytes; thus, JAK2 is a critical signaling event controlling integrin triggering by chemokines also in neoplastic B-cells; b- in keeping with the previous point, JAK2 controls RhoA activation by chemokines in normal as well as CLL B-lymphocytes and this explains the pro-adhesive role of JAK2; c- JAK2 tyrosine phosphorylation responsible for kinase activation is preserved in normal versus CLL B-lymphocytes, thus possibly suggesting that no mutations negatively affecting JAK2 functionality occur in B-CLL; d- analysis of 41 B-CLL patients did not evidence JAK-independent phenotypes, suggesting that, in spite of disease heterogeneity, JAK2 regulatory role is conserved in B-CLL.

JAK PTKs have been originally implicated in cytokine signaling [18], thus participating to regulation of proliferation, anti-apoptotic and pro-survival pathways. Many evidences suggest the role of JAKs in cancer development. For instance, a chromosomal translocation resulting in the constitutively activated fusion protein TEL-JAK2 was identified in a human T-cell lymphoblastic leukemia [19]. Moreover, mutation in the *JAK2* gene was reported in about 80% of patients with a diagnosis of myeloproliferative neoplasms [20]. Such a mutation determines the substitution of a phenylalanine for valine 617 (V617F) and is believed to disrupt the auto inhibitory activity of the JAK2 pseudokinase domain. Importantly, it was shown that myeloproliferative neoplasms can be initiated from a single hematopoietic stem cell expressing V617F mutated JAK2 [21]. More recently, activating mutations in JAK2 exon 12, encoding a region close to the pseudokinase domain, were identified in JAK2 (V617F)-negative patients [22]. Finally, JAK2 activating mutations have been also reported in acute lymphoblastic leukemia [23]. All these mutations are likely responsible for hypersensitivity to cytokine triggering and cytokine-independent growth [22]. Accordingly, signaling pathways involved in cell proliferation and survival, such as STAT, MAPK, and PI3K-AKT, are up regulated in hematopoietic cells expressing JAK2 (V617F) [22].

Very recently, the JAK2 V617F mutation was detected in two B-cell chronic lymphocytic leukemia patients without coexisting Philadelphia chromosome-negative myeloproliferative neoplasms [24], thus suggesting that, although rare, this JAK2-activating mutation may occur also in B-CLL. In our study, a genetic analysis was not performed, so we cannot totally exclude that a JAK2 V617F mutation was present in one or more patients we analyzed. However, the data may suggest that JAK2 was not mutationally activated in our group of patients. Indeed, considering the critical role of JAK2 in integrin activation, if a V617F mutated JAK2 was expressed, we should have detected, in absence of any chemokine stimulation, higher spontaneous cell adhesion and constitutive LFA-1 high affinity state in B-CLL subjects with respect to healthy donors. Moreover, at biochemical level, we should have observed a higher basal level of JAK2 auto-phosphorylation on the critical 1007–1008 tyrosine residues. However, no difference was detected in B-CLL patients compared to healthy subjects, thus suggesting that JAK2 was not constitutively activated in our analysis. On the other hand, the capability of CXCL12 to activate JAK2 and the inhibitory effect of JAK2 inhibitors on integrin affinity up regulation and mediated rapid adhesion clearly suggest that no mutations negatively affecting JAK2 kinase activity occur in B-CLL. This is also in keeping with the ability of JAK2 to regulate RhoA activation by CXCL12 in CLL B-lymphocytes. Thus, JAK2 is functional and normally regulated by chemokines in CLL B-lymphocytes, at least in the group of studied patients and, differently from Rac1 and PIP5K1C [13], appears indispensable for CXCL12-triggered integrin activation. However, considering the heterogeneity of the disease and the presence of particularly aggressive transformation forms of B-CLL, such the Richter's syndrome, it will be of great interest to perform a systematic genetic analysis of JAK2 mutations in B-CLL.

Altogether, our data identify JAK2 as a critical regulator of B-lymphocyte adhesion by CXCL12 and suggest that inhibition of JAK2 could be an innovative therapeutic approach to B-CLL treatment. Several JAK inhibitors have been generated with some already approved, such as Ruxolitinib (psoriasis, myelofibrosis and rheumatoid arthritis) and Tofacitinib (psoriasis and rheumatoid arthritis), or in clinical trials such as Baracitinib (rheumatoid arthritis), CYT387, (myeloproliferative disorders), Lestaurtinib (acute myelogenous leukemia), Pacritinib (lymphoma and myeloid malignancies), TG101348 (myelofibrosis). Notably, the BTK inhibitor Ibrutinib has emerged in recent years as a very promising drug in B-CLL treatment, although few patients show mechanisms of resistance due to C481S mutation of BTK sequence preventing the irreversible binding of Ibrutinib to BTK. Thus, JAK inhibition could be an interesting alterative in those cases where BTK inhibition is ineffective. Furthermore, BTK was very recently suggested to be a downstream effector of CXCL12 [25], although its role in integrin activation by chemokines was not clarified. Thus, it will be significant to study the involvement of BTK in rapid integrin triggering by chemokines and to test the possible functional interplay between BTK and JAKs to define the possibility of combined treatments. Considering the relevant complexity of signaling networks triggered by chemokines, a systematic analysis of signaling events involved in chemokine-triggered adhesion in CLL B-lymphocytes can be an appealing strategy to devise new treatments to relieve disease symptoms and to block its progression.

## MATERIALS AND METHODS

### Reagents

Human E-selectin/Fc, human ICAM-1/Fc and human CXCL12 were from R&D Systems (R&D Systems, Minneapolis, MN, USA); fluorescein isothiocyanate (FITC) goat secondary antibody to mouse was from Sigma (Sigma-Aldrich, St. Louis, MO, USA); KIM127 mouse monoclonal antibody was from American Type Culture Collection (ATCC, Rockville, MD, USA); 327C and A mouse monoclonal antibodies were kindly provided by dr. Kristine Kikly (Eli Lilly and Co., Indianapolis, IN, USA); Tyrphostin AG490 and rabbit polyclonal anti-actin antibody were from Sigma; rabbit monoclonal anti-JAK2 (D2E12), was from Cell Signaling Technology (Danvers, MA, USA); siRNAs (ON-TARGETplus SMARTpool) were from Thermo Fisher Scientific (Fremont, CA, USA).

### Isolation of B-lymphocytes from healthy subjects and B-CLL patients

Normal and CLL B-lymphocytes were isolated from PBMCs after blood separation on Ficoll Paque Plus (GE Healthcare, Little Chalfont, UK) and purification by negative selection (EasySep™ Human B Cell Enrichment Kit; Stemcell Technologies, Vancouver, BC, Canada). Purity of B-lymphocyte preparations was evaluated by flow cytometry with anti-CD19 mAb (BD Biosciences, San Jose, CA, USA). The study involved a total of 41 patients with a diagnosis of B-CLL compared to normal B-cells. The diagnosis of B-CLL was made upon clinical and laboratory parameters, including the complete blood cell count, peripheral blood smear and immunophenotype of the circulating lymphoid cells, according to the current guidelines and fulfilling diagnostic and immunophenotypic criteria for common B-CLL [26] at the hematology section of the Department of Clinical and Experimental Medicine, University of Verona. B-CLL patients have been selected for complete absence of any previous treatment. Samples were obtained with informed consent and the approval of the Ethics Committee. Blood samples from B-CLL patients contained CD5 positive cells ranging from 30% to 95% with an average of 76 ± 14% (see Table 1). Normal and CLL B-lymphocytes were plated at 5 × 10^6^/ml in RPMI + 2 mM Glutamine + 10% FBS for 3 h before treatment with inhibitors or Trojan peptides.

**Table 1:** List of B-CLL patients involved in the study

Patient	% CD5/CD19	IgVH	CD38	Age	Gender
**1**	75	NM	P	71	M
**2**	60	M	P	52	M
**3**	86	ND	N	43	F
**4**	95	NM	P	73	M
**5**	75	NM	P	68	M
**6**	88	ND	P	76	M
**7**	85	ND	P	75	M
**8**	86	ND	N	63	F
**9**	88	ND	P	81	F
**10**	60	M	N	63	M
**11**	95	NM	N	72	F
**12**	91	M	N	49	M
**13**	95	ND	P	76	F
**14**	72	NM	N	73	M
**15**	80	ND	N	85	F
**16**	80	ND	N	66	M
**17**	75	ND	N	55	F
**18**	86	ND	N	85	M
**19**	85	ND	P	60	F
**20**	50	ND	P	64	M
**21**	52	M	N	62	M
**22**	60	ND	P	73	M
**23**	80	M	N	58	F
**24**	54	ND	P	71	F
**25**	90	ND	N	89	M
**26**	90	NM	P	53	F
**27**	70	ND	N	67	M
**28**	77	ND	N	90	F
**29**	70	ND	N	68	M
**30**	30	ND	N	64	F
**31**	72	ND	N	64	M
**32**	65	ND	N	77	F
**33**	90	ND	N	84	F
**34**	88	ND	N	77	F
**35**	65	ND	P	44	F
**36**	72	ND	N	73	F
**37**	95	ND	P	72	M
**38**	76	M	N	65	M
**39**	70	ND	N	84	F
**40**	87	NM	N	87	F
**41**	68	ND	N	79	F

41 patients with a diagnosis of B-CLL were involved; NM not mutated; M mutated; ND not detected; P positive (>30% on B-cells), N negative (<30% on B-cells).

### Static adhesion assay

B-lymphocytes were suspended at 5 × 10^6^/ml in standard adhesion buffer (PBS + 10% FBS + Ca^2+^ 1 mM + Mg^2+^ 1 mM, pH 7.2). Adhesion assays were performed on 18-well glass slides coated with human ICAM-1 or VCAM-1, 1 μM in PBS. 20 μl of cell suspension were added to the well and stimulated at 37°C with 5 μl of CXCL12, 0.5 μM final concentration, for 120 sec. After washing, adherent cells were fixed in glutaraldehyde 1.5% in ice-cold PBS and counted by computer-assisted enumeration.

### Underflow adhesion assay

B-lymphocytes were suspended at 1.5 × 10^6^/ml in standard adhesion buffer. Cell behavior in underflow conditions was studied with the BioFlux 200 system (Fluxion Biosciences, South San Francisco, CA, USA). 48-well plate microfluidics were first co-coated overnight at RT with 2.5 μg/ml human E-selectin together with 10 μg/ml human ICAM-1 or with VCAM-1 alone in PBS. Immediately before use, microfluidic channels were washed with PBS and then coated with 4 μM CXCL12 in PBS for 3 h at RT and the assay was done at wall shear stress of 1 dyne/cm^2^. After extensive washing of microfluidics with adhesion buffer, the behavior of interacting lymphocytes was recorded on digital drive with a fast CCD videocamera (25 frames/s, capable of 1/2 subframe 20 ms recording) and analyzed subframe by subframe. Single areas of 0.2 mm^2^ were recorded for at least 60 sec. Interactions of 20 msec or longer were considered significant and scored. Lymphocytes that remained firmly adherent for at least 10 sec, thus including also events of adhesion stabilization, were considered fully arrested and scored. Rolling interacting and arrested cell behaviors were automatically detected and quantified with BeQuanti (http://www.embeddedvisionsystems.it/solutions/medical-imaging).

### Measurement of LFA-1 affinity states

B-lymphocytes, suspended in standard adhesion buffer at 2 × 10^6^/ml, were briefly pre-incubated with 10 μg/ml of KIM127 or 327A mAbs and then stimulated for 120 sec with 0.5 μM CXCL12 (final concentration) under stirring at 37°C. After rapid washing, cells were stained by FITC secondary polyclonal antibody and analyzed by cytofluorimetric quantification.

### JAK2 activation

JAK2 activation was measured by ELISA kit (JAK2 pYpY1007/1008; Life Technology, Carlsbad, CA, USA), following manufacturer's instructions. Briefly, 5 × 10^6^ cells were treated and stimulated as indicated and then lysed at 4°C. Cell lysates were tested for pYpY1007/1008 JAK2 and colorimetric signals were detected by a plate reader (Victor™ X5 Multilabel Plate Reader, Perkin Elmer, Waltham, MA, USA).

### RhoA activation

RhoA activation was measured by using commercial kit (G-LISA RhoA Activation Assay Biochem Kit; Cytoskeleton, Denver, CO, USA), following manufacturer's instructions. Briefly, 5 × 10^6^ cells were treated and stimulated as indicated and then lysed at 4°C. Cell lysates were tested for GTP loaded RhoA and colorimetric signals were detected by a plate reader (Victor™ X5 Multilabel Plate Reader, Perkin Elmer).

### Gene silencing of JAK2 by siRNA

siRNAS were provided as a premixed pool (SmartPool) [16]. Silencing was performed in normal B-lymphocytes by nucleoporation, using the Amaxa Nucleofector [13]. The efficacy of gene silencing was evaluated by immunoblotting after 24 h and 48 h. Intensities of band signals were quantified by densitometric analysis (Quantity One, Bio-Rad) by using ImageQuant Las4000 (GE Healthcare Life Science).

### Statistical analysis

Results are expressed as mean ± standard deviation (SD). Statistical analysis was carried out by calculating mean and SD between different experiments. Significance was calculated by two-tailed Student's *t*-test (applied for underflow experiments) or one-way analysis of variance (ANOVA) followed by *post hoc* Dunnett's multiple comparisons test at the 95% confidence level. A *p* < 0.05 was considered statistically significant. All statistical analyses were performed using GraphPad Prism 6 (GraphPad Software, Inc., San Diego, CA, USA).

## SUPPLEMENTARY FIGURE


