# Supplementary material for: JAK2 tyrosine kinase mediates integrin activation induced by CXCL12 in B-cell chronic lymphocytic leukemia
Source: Oncotarget. 2015 Sep 10;6(33):34245–57. doi: 10.18632/oncotarget.5196 (PMC4741449; doi:10.18632/oncotarget.5196)
Supplement: Supplementary file 1 [file oncotarget-06-34245-s001.pdf]

SUPPLEMENTARY FIGURE

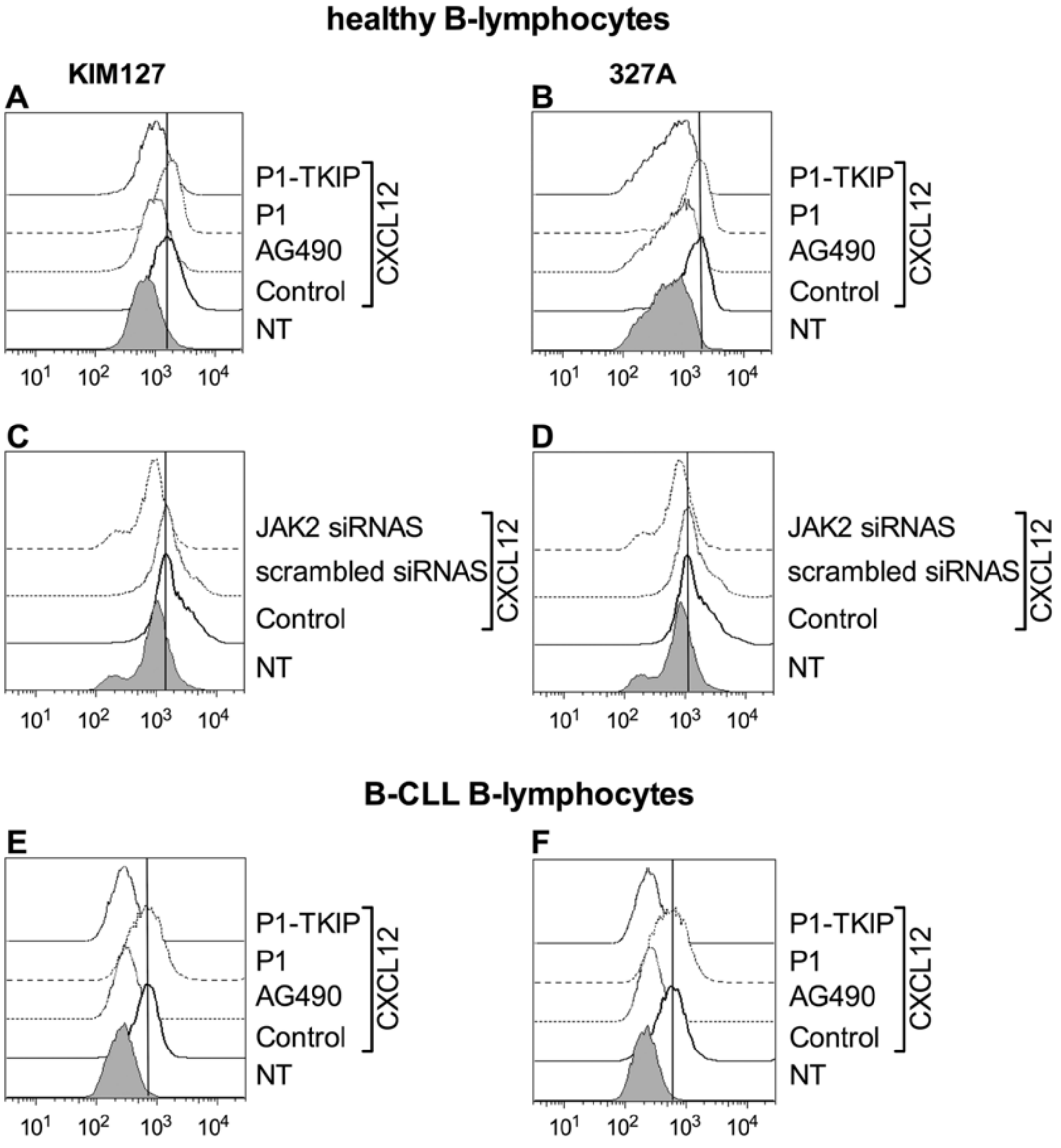

Supplementary Figure S1: Representative flow cytometry data. Results are presented as histograms of fluorescence intensity in log scale. Histograms on the left represent cells stained with KIM127 antibody; histograms on the right represent cells stained with 327A antibody. **A–B.** B-lymphocytes from healthy donors were treated for 1 h with vehicle (NT and Control, grey filled and bold black line histograms, respectively), AG490 100 µM (dashed line histogram), P1 (long dashed line histogram) or P1-TKIP 40 µM (black line histogram), and stimulated with CXCL12 0.5 µM for 120 sec. **C–D.** Cells were not treated with siRNAs (NT and Control, grey filled and bold black line histograms, respectively), nucleoporated with a pool of 4 scrambled (dashed line histogram) or JAK2-specific (long dashed line histogram) siRNAs and kept in culture for 48 h before staining with KIM127 or 327A antibodies. **E–F.** B-lymphocytes from B-CLL patients were treated as in (A–B).
